# Biomimetic optimization research on wind noise reduction of an asymmetric cross-section bar

**DOI:** 10.1186/s40064-016-2857-2

**Published:** 2016-08-02

**Authors:** Yingchao Zhang, Weijiang Meng, Bing Fan, Wenhui Tang

**Affiliations:** 1 State Key Laboratory of Automotive Simulation and Control, Jilin University, Changchun, 130022 China; 2 Key Laboratory Bionic Engineering, Ministry of Education, Jilin University, Changchun, 130022 China

**Keywords:** Biomimetics, Asymmetric section geometry, Non-smooth characteristics, Computational aeroacoustics, Noise level

## Abstract

**Background:**

In this paper, we used the principle of biomimetics to design two-dimensional and three-dimensional bar sections, and used computational fluid dynamics software to numerically simulate and analyse the aerodynamic noise, to reduce drag and noise.

**Methods:**

We used the principle of biomimetics to design the cross-section of a bar. An owl wing shape was used for the initial design of the section geometry; then the feathered form of an owl wing, the v-shaped micro-grooves of a shark’s skin, the tubercles of a humpback whale’s flipper, and the stripy surface of a scallop’s shell were used to inspire surface features, added to the initial section and three-dimensional shape.

**Results:**

Through computational aeroacoustic simulations, we obtained the aerodynamic characteristics and the noise levels of the models. These biomimetic models dramatically decreased noise levels.

## Background

In 1952, British scholar James Lighthill used “acoustic analogy” to derive Lighthill’s equation from the Navier–Stokes equations; Lighthill’s equation describes the noise of moving airflow (Lighthill 1952). In 1969, Ffowcs-Williams and Hawkings expanded the Curle analogy to consider the effects of a moving solid boundary on sound, and introduced the Ffowcs-Williams–Hawkings (FW–H) equation (Williams and Hawkings 1969). In 1998, Cox and Brentner studied vortex shedding and noise radiation around a cylinder (Cox et al. 1998), and found two-dimensional (2D) computational fluid dynamics (CFD) noise prediction to be fast and convenient. In 2002, Myunghan and Jeonghan studied the aerodynamic noise of a rack beam that featured an asymmetric section bar (Myunghan et al. 2002). In 2004, Perot and Auger studied the noise generated by low-Mach-number flows around a cylinder and a wall-mounted half-cylinder (Pérot et al. 2004), and verified the resolution growth of the FW–H equation by using a higher order time algorithm. In 2012, Cox and Rumsey studied the computation of sound generated by viscous flow over a circular cylinder (Cox et al. 2012). In 2003, Yu Chao was able to predict 2D parallel shear layer sound, by using the integral method (Yu and Li 2003); they showed that their integral solution agreed well with a computational aeroacoustics numerical solution. In 2008, Sun Shaoming and Ren Luquan studied the noise reduction mechanism of the non-smooth leading edge of an owl’s wing (Ren et al. 2008); they combined biomimetics with aerodynamic noise optimization. In 2011, Shi Lei, Zhang Chengchun produced a noise reduction optimization of the NACA0018 aerofoil model (Shi et al. 2011), and found that a saw tooth leading and trailing edge could effectively reduce noise at high speeds. In 2014, Zhang Chengchun and Wang Wenqiang studied the aerodynamic noise reduction of a cylindrical rod with a biomimetic wavy surface. They found that the aerodynamic noise of the circular cylinder was reduced (Zhang et al. 2014).

This paper adds biomimetic features to the design of an asymmetric bar and explores the drag and noise reduction effects of various surface features. To ensure the accuracy of the simulation results, we constructed a three-dimensional (3D) model for verification.

## Methods

 The commercial CFD software StarCCM+ by CD-Adapco was used for the 2D and 3D aeroacoustic simulations (Zhang 2011). The K-Omega-SST turbulence model was used in the transient 2D simulations. By using the FW–H model, we could predict the middle and far field noise sources and observe the noise reduction effect of each design scheme. For the computational methods, we used a FW–H acoustic model; this assumes that the boundary surface can penetrate the flow, creating a discontinuity. The FW–H control equation can be written as:

1 $$\left( {\frac{{D_{\infty }^{2} }}{{D_{t}^{2} }} - c_{\infty }^{2} \frac{{\partial^{2} }}{{\partial x_{i}^{2} }}} \right)[(\rho - \rho_{\infty } )H(f)] = \frac{{\partial^{2} }}{{\partial x_{i} \partial x_{j} }}[T_{ij} H(f)] - \frac{\partial }{{\partial x_{i} }}[F_{\text{i}} \delta (f)] + \frac{\partial }{{\partial_{t} }}[Q\delta (f)]$$

2 $$T_{ij} = \rho (u_{i} - U_{i}^{\infty } )(u_{j} - U_{j}^{\infty } ) + (p - c_{\infty }^{2} (\rho - \rho_{\infty } ))\delta_{ij} - \tau_{ij}$$

where:

3 $$F_{i} = - \left[ {\rho \left( {u_{i} - 2U_{i}^{\infty } } \right)u_{j} + \rho_{\infty } U_{i}^{\infty } U_{j}^{\infty } + p\delta_{ij} - \tau_{ij} } \right]\frac{\partial f}{{\partial x_{j} }}$$

4 $$Q = \left[ {\rho u_{i} - \rho U_{i}^{\infty } } \right]\frac{\partial f}{{\partial x_{i} }}$$

Equation 1 has a clear physical meaning, the three right-hand parts of the equation represent the main types of acoustic radiation source. The first represents the turbulent shear stress of the fluid itself, and takes the form of a quadrupole. The second represents the divergence of an unsteady force on an interface, and takes the form of a dipole. The third includes the quality of unsteady flow entering into the fluid, its action is unrelated to a monopole. Through the FW–H method, time and space were discredited by an appropriately small time step and a relatively fine mesh, enabling second-order prediction of far field noise.

In the 3D simulations, the K-Omega-SST turbulence model was first used for steady simulation, and the model of Curle and Proudman was used to predict noise sources. We then used the K-Omega-SST detached eddy simulation (DES) model for transient simulation. After getting the pressure from each monitoring point, we obtained the sound pressure level values through a fast Fourier transform. Thus we could compare the advantages and disadvantages of each design scheme.

We use the finite volume method as the spatial discrete control equation, the Computational domain is divided into volume meshes, and there is no overlap of a control volume around each volume mesh, the spatial discrete equations can be solved from each control volume integral. We use implicit time discretization scheme, each unknown discrete equations are coupled together. After determining the time step, we should solve the coupled linear equations of each time step.

### 2D models

In 2003, Pietro Catalano and Meng Wang numerically simulated the flow around a circular cylinder at high Reynolds numbers (Catalano et al. 2003), and established the associated drag coefficient; this paper provides good validation data for our simulation results. We ran a steady simulation of a circular cylinder at a Reynolds number of 1.32 × 10^5^, and found the drag coefficient to be 1.026; the experimental result was 1.04 for the same Reynolds number. The error range was thus limited to 2 %, validating our simulation result.

**Fig. 1:**
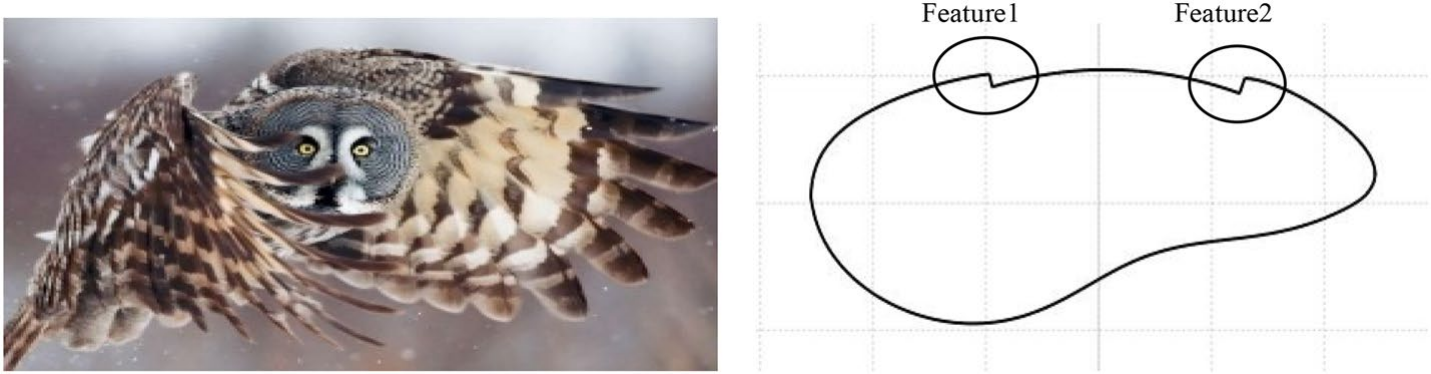
Owl’s wings (*left*), and the biomimetic asymmetric two-dimensional basic model 1-0 (*right*)

To select the asymmetric model we referred to the acoustical properties of owl wings; we set the cross-sectional shape of the 2D cylinder accordingly (Fig. 1).

**Fig. 2:**
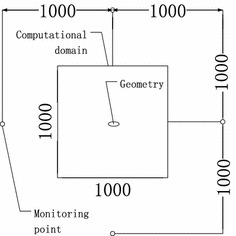
Computational domain and monitoring points

The cross-sectional length and width of the asymmetric circular section were 50 and 20 mm, respectively; the shape of the computational domain (Fig. 2), was rectangular. The boundary was set as free, the free stream velocity was set to 50 m/s, the Mach number was set to 0.144. There were four monitoring points which were set at the right, left, top, and bottom parts of the geometry, each 1000 mm from the 2D model.

For the noise calculation, we used an implicit transient solution. The surface of the FW–H for acquiring pressure signals was set to be the surface of the asymmetric model. The maximum target frequency was 5000 Hz, the time step was 10 µs, the time was set as a second order discretization, the largest internal iteration step was 10, the largest computing time was 0.15 s, and the computing time of the FW–H solver was 0.12 s. Averages were taken over 20,000 timesteps, the sample frequency was five times the maximum frequency. The FW–H solver computed over 0.03 s and the averaging number of the spectra was 600, the maximum target frequency was 10,000 Hz; based on the Nyquist frequency law, when the sample frequency is double the maximum frequency, it is sufficient.

The study found that the drag and noise of the 2D cylinder were large; the maximum drag was 43 N, the peak noise was 110 dB. Therefore, noise reduction and drag reduction of the cylindrical bar are necessary.

**Fig. 3:**
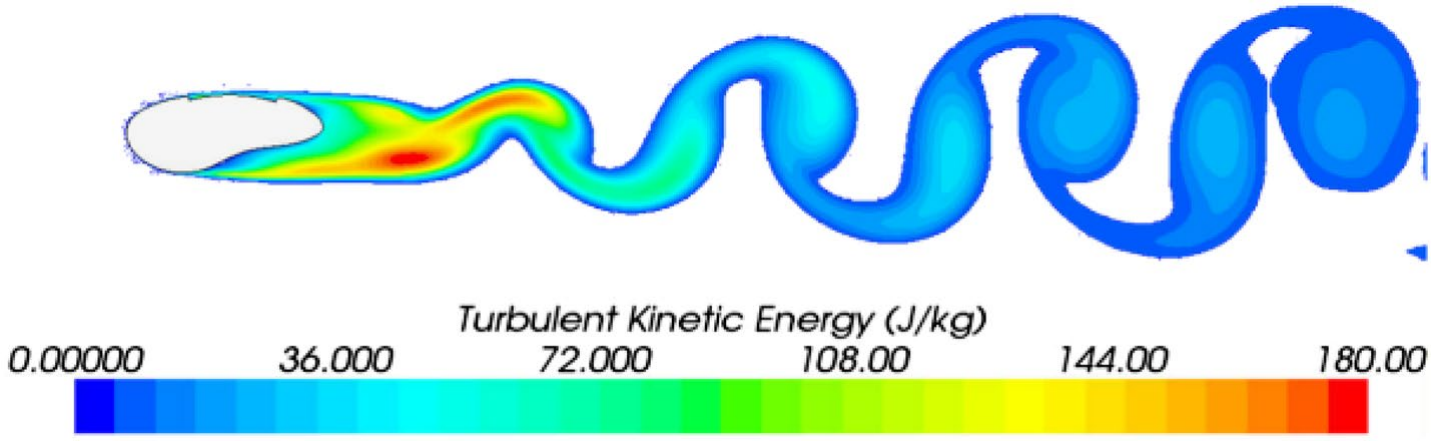
Turbulent kinetic energy section of model 1-0

The numerical simulation of the 2D asymmetric model yielded the turbulent kinetic energy (Fig. 3).

**Fig. 4:**
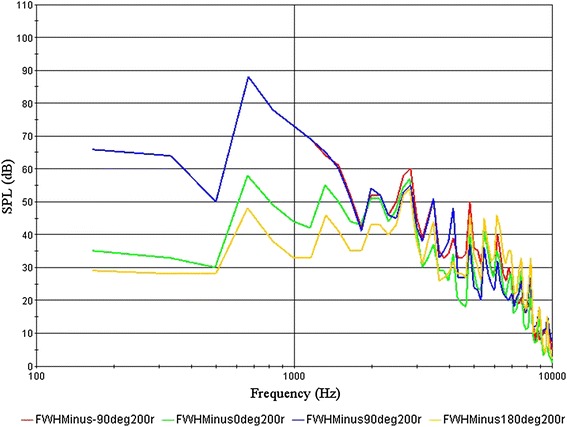
Sound pressure level curve of model 1-0

By analysing the results of model 1-0, we found that the maximum drag was 12.41 N and the maximum noise was 88 dB. The sound pressure level curve for the monitoring positions is shown in Fig. 4.

**Fig. 5:**
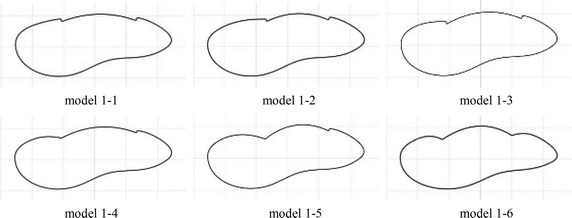
Improved models

Through the analysis of the results, we found that the maximum drag was still large, but the maximum noise was quieter. To further optimize the structure, we paid more attention to drag reduction. To optimize the basic structure, we adjusted the positions of features 1 and 2, to produce different foundation models (Fig. 5).

Compared with the basic model, model 1-1 and model 1-3 moved feature 1 in the downward direction; model 1-2 moved feature 1 downwards and backwards; model 1-4, when compared with model 1-2, changed the shape of feature 1 from outer convex to inner concave; model 1-5, when compared with model 1-3, changed the shape of feature 1 from outer convex to inner concave; model 1-6, when compared with model 1-3, changed the shapes of features 1 and 2 from outer convex to inner concave.

From Table 1, the drag reduction and noise reduction of all of the modified models are improved when compared with the original model. The most significant improvement is model 1-5 with a reduction of maximum drag of 17.9 %, and a maximum noise reduction of 11 dB. So we selected models 1-3 and 1-5 for the addition of non-smooth characteristics.

**Table 1:** Maximum resistance and maximum noise of the improved models

Model number	Maximum drag/N	The reduction rate of max drag (%)	Maximum noise/dB
Model 1-0	12.41	–	88
Model 1-1	11.15	10.1	82
Model 1-2	10.82	12.8	82
Model 1-3	10.43	15.9	80
Model 1-4	10.46	15.7	80
Model 1-5	10.18	17.9	77
Model 1-6	9.26	25.4	88

**Fig. 6:**
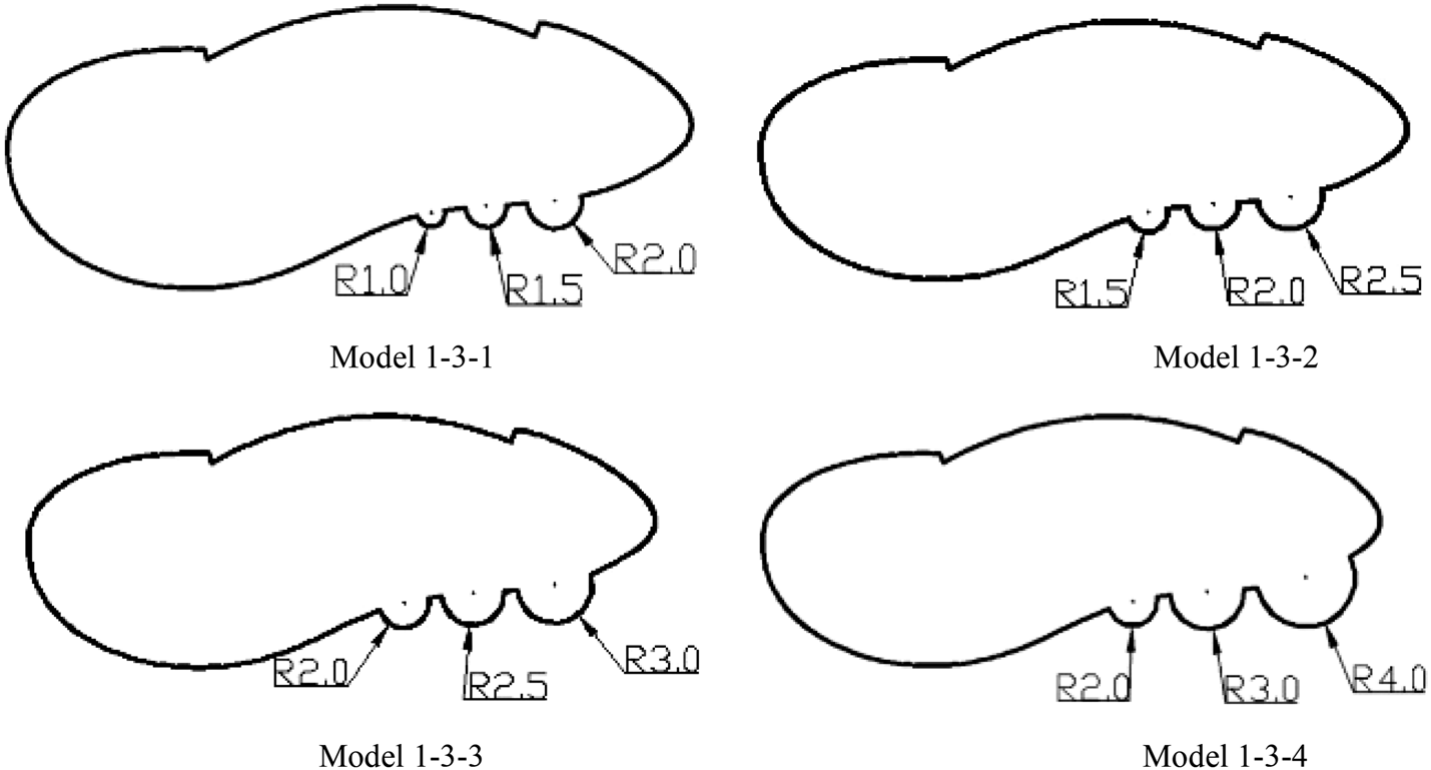
Optimization models

Based on the basic asymmetric optimization structure 1-3, by referring to the humpback whale flipper tubercles, we selected a semicircle as a non-smooth feature, and configured the following models to study the drag and noise reduction; the maximum values are shown in Table 2, the maximum drag and noise were significantly lower. As shown in Fig. 6, the largest reduction of the maximum noise came from model 1-3-2 with a maximum radius of 2.5 mm, the maximum noise reduced from 42 to 38 dB; the maximum drag reduction rate was 24.9 %, the maximum drag was 7.83 for model 1-3-3 in which the largest radius was 3 mm.

**Table 2:** The maximum drag and noise of the optimized models

Model number	Maximum drag/N	The reduction rate of max drag (%)	Maximum noise/dB
Model 1-3-1	8.82	15.4	49
Model 1-3-2	8.12	22.1	38
Model 1-3-3	7.83	24.9	43
Model 1-3-4	8.52	18.3	80

The biomimetic asymmetric bar had achieved effective results both on noise and drag reduction; we chose model 1-3-2 as the optimal model for further research.

**Fig. 7:**
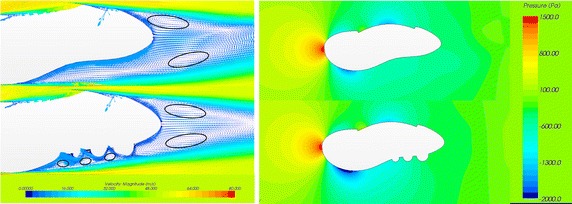
Velocity vector cross section (*left*) and pressure distribution (*right*) in the cross section of the 1-3 model (*upper*) and the optimized models (*lower*)

By comparing the velocity vector diagram of the original model 1-0 and the optimal model 1-3-2 (Fig. 7), we found two obvious eddy currents on the trailing edge behind the model. The three semicircles, caused three small vortices at the bottom of the model that affected the turbulence intensity of the airflow. Because of the flow interference at the bottom, the two eddy currents present a symmetrical wake, when compared with the original model which has a tandem distribution state. The optimised case maintained a symmetrical state, but the tandem eddy current of the original model caused the downstream flow to cyclically change, generating a vortex street and producing a louder noise.

Comparing the stress distribution near the two models (Fig. 7), we found that the pressure value of the model with the semicircles was smaller than that of the original model, and the front pressure of the semicircle model was small, producing reverse thrust and reducing drag around the three semicircles.

To further verify the drag and noise reduction capabilities of the semicircle model, we also added non-smooth semicircles to the models. We achieved a maximum drag and noise of 8.90 N and 39 dB, respectively. Compared with the basic model 1-5, the resistance and noise values were much smaller.

Thus the non-smooth semicircle model had a very good effect on noise and drag reduction.

### Effect of velocity to noise

**Fig. 8:**
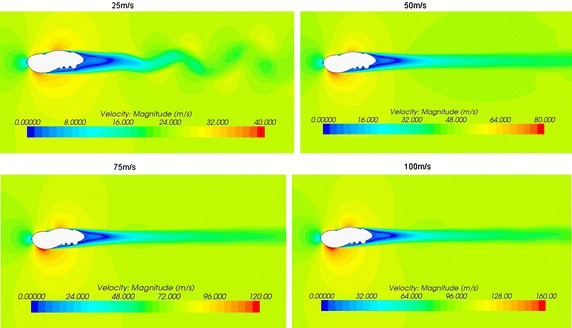
Velocity magnitude of model 1-3-2 at different velocities

**Fig. 9:**
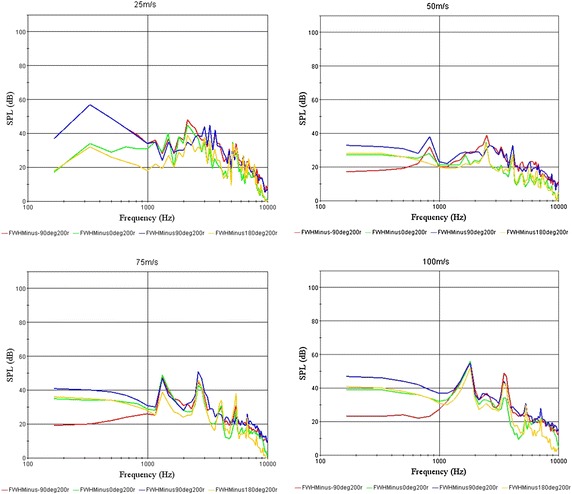
Sound pressure level curves at different velocities

To achieve the applied range of the non-smooth characteristics, we performed numerical simulations of optimal model 1-3-2 at velocities of 25 m/s, 50 m/s, 75 m/s and 100 m/s; we obtained the velocity magnitude (Fig. 8) and the sound pressure level curves (Fig. 9) for model 1-3-2.

By comparing the sound pressure level curves of optimal model 1-3-2 with the original model 1-0 (Fig. 4), we found that when the speed was low, the low frequency noise is louder; as the speed increased, the peak noise value moved towards the high-frequency region. With increasing speed, the noise reduction effect of the biomimetic asymmetric bars was evident, but it was not obvious at low speeds. So compared to other bars, the operating conditions of the asymmetric shape is more suitable for high-speed applications. At low speeds the noise of the bar was low; the noise reduction on asymmetric bars was thus meaningful.

### 3D models

#### Establishment of 3D models

**Fig. 10:**
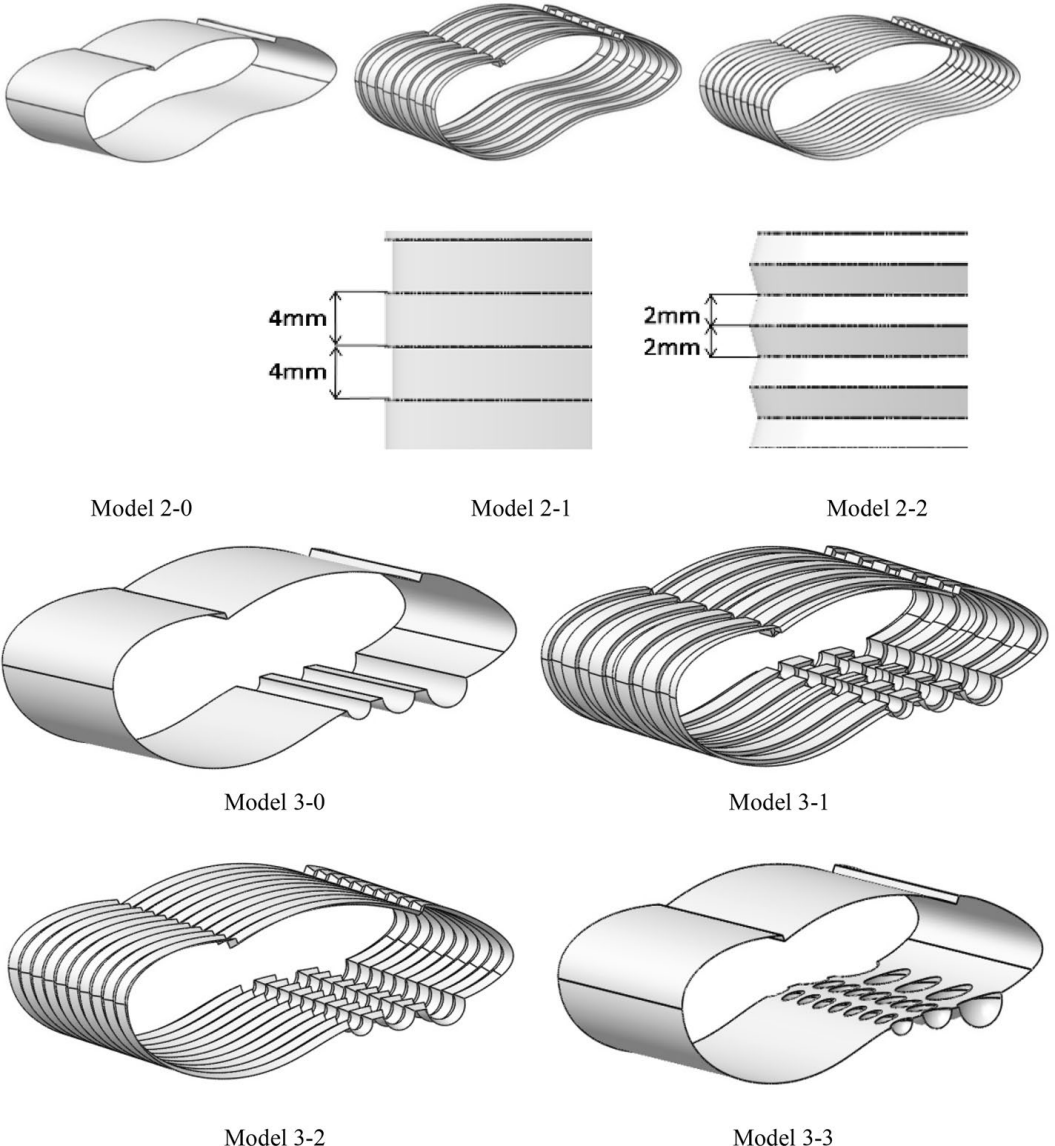
Three-dimensional models

After finding the optimised 2D shape, we stretched the asymmetric cross-section 2D Model to a 40-mm-thick 3D Model. To verify the noise reduction effect of the non-smooth characteristics, we referred to the parallel arranged V-shaped micro groove structures seen on the surface of shark skin, and the striped U-shaped surface structures seen on scallops. We added the U and V grooves on to the basic 3D model (Fig. 10).

**Fig. 11:**
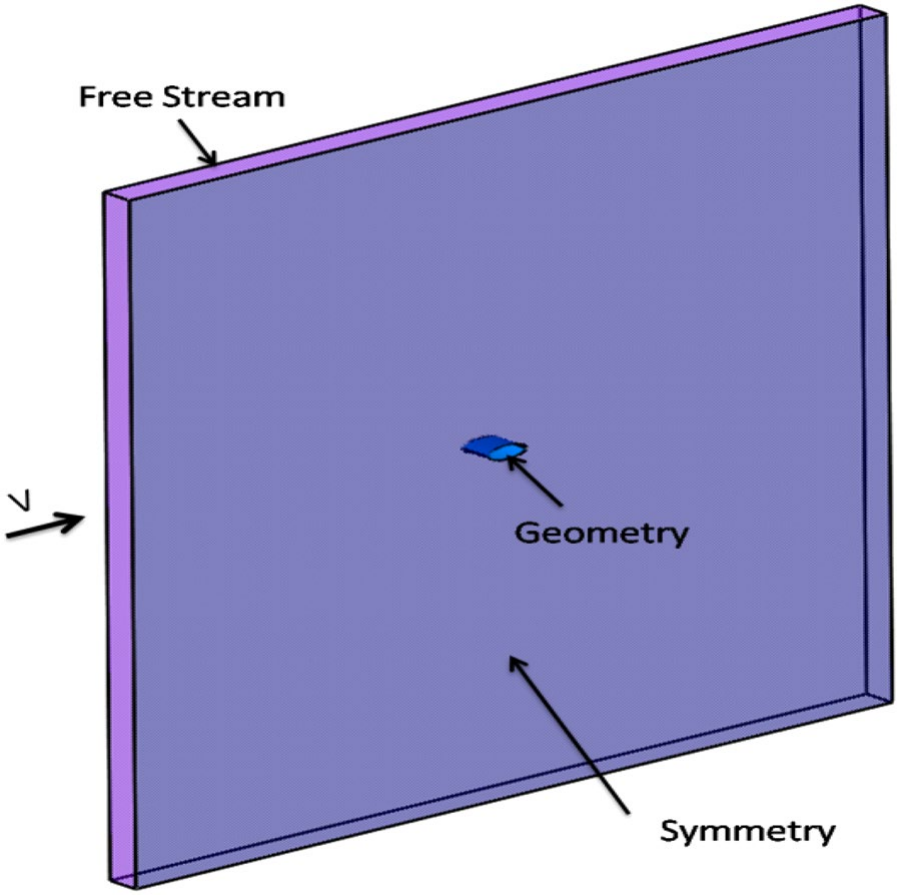
Computational domain of 3D models

The 3D computational domain was obtained by directly using the 2D domain (Fig. 11). The boundaries were set to free stream, and the free flow speed was 50 m/s; the two sides were set as symmetry planes. Monitoring locations were placed around the model; the top and the bottom locations were at a distance of 15 mm from the origin, the front was 25 mm, the back was 35 mm.

DES models require a finer grid, so on the basis of the 2D grid, we refined the volume mesh by adding some more refinement blocks. The first layer of the boundary layer mesh was set to 5 µm, with a total of 15 layers and total thickness of 1 mm.

The 3D calculation used a K-Omega-SST model for the steady-state simulation, and used the calculated results as the initial value of the transient simulation. At the same time, it used a broadband noise source model to predict the noise source. The broadband noise source models are intended to be used as tools for qualifying a volume mesh before a transient acoustic analysis; we used the Curle and Proudman noise source models. The Curle acoustic power model computes the sound that dipole sources generate, The Proudman acoustic power model estimates the local contribution of the quadrupole sources. The steady-state analysis process finds the grid area that requires encryption, and ensures that the whole model surface Y+ values are below 1. In this paper, the frequency of the 3D calculation was set to 5000 Hz.

Before the start of the transient calculation, the transient parameters were set. The frequency of the 3D calculation was 5000 Hz, and the cycle was 200 µs. The length of a time step was set to 50 µs, and the simulation of the computation time was 0.1 s, the number of iterations for every time step was 10. After setting the calculation parameters, the surface pressure data was automatic exported after the calculation of each time step. When the calculation completed, we obtained the drag value, and the pressure data of the monitoring points which were used to get the sound pressure level curves. Through a Fourier transform of the automatic export pressure data, we obtained the flow field noise distribution.

#### 3D numerical simulation

**Fig. 12:**
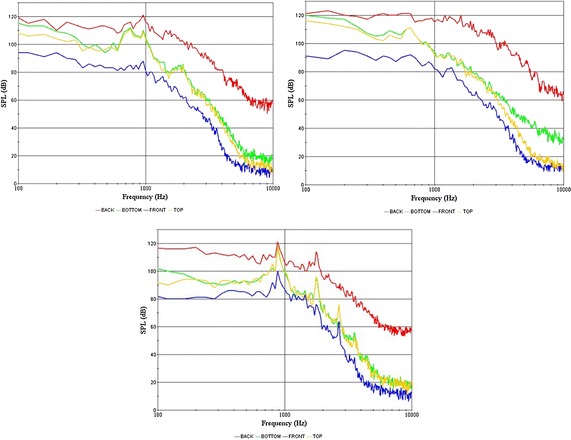
Sound pressure level curves of the three-dimensional models (Model 3-0, Model 3-1, Model 3-3)

**Fig. 13:**
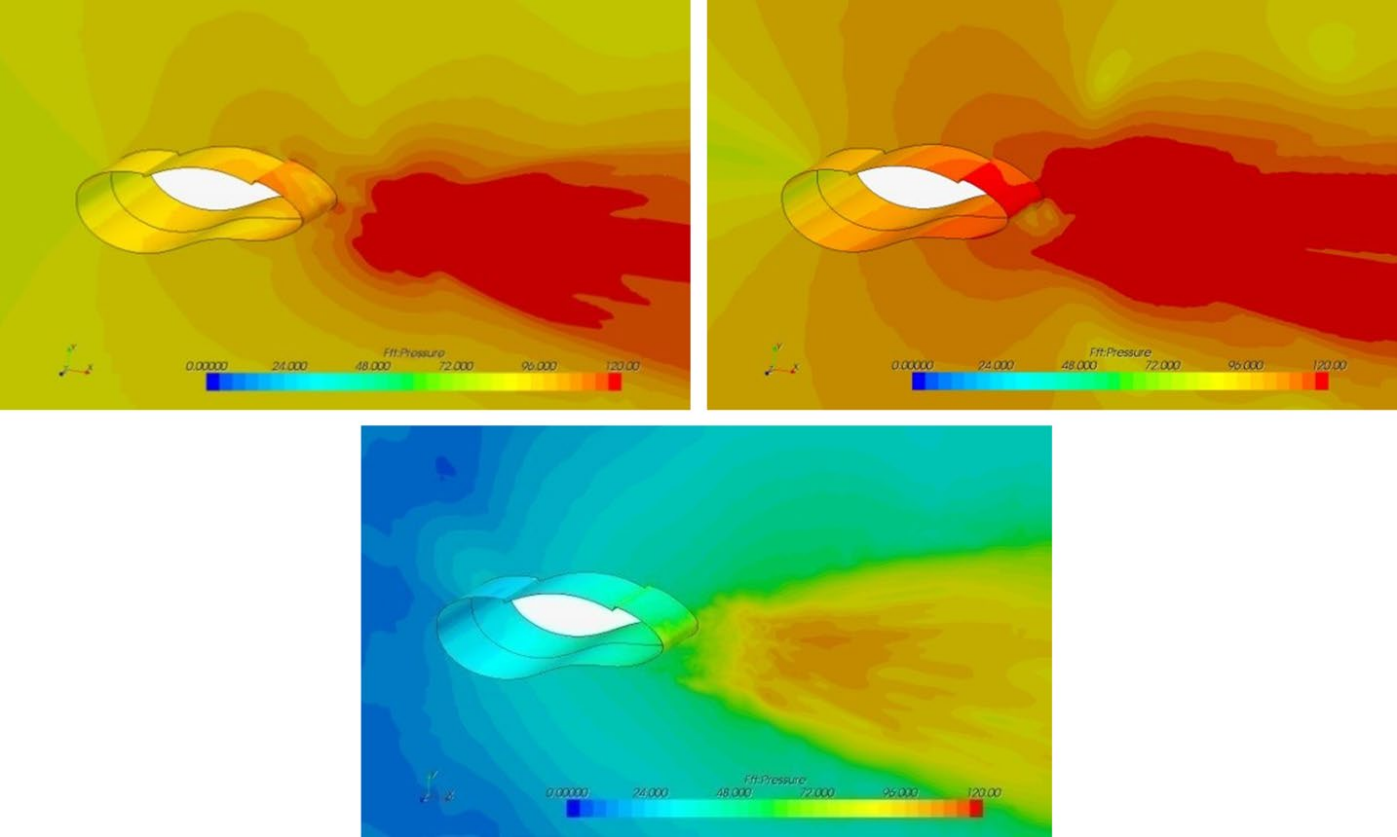
Sound pressure diagrams at 500 Hz, 1000 Hz and 5000 Hz

From the simulations, we obtained the sound pressure level curves for each of the models, and obtained the maximum noise, as shown in Fig. 12 and Table 3. Meanwhile, we obtained the sound pressure diagrams at 500 Hz, 1000 Hz and 5000 Hz, as shown in Fig. 13.

**Table 3:** Maximum noise of the asymmetric 3D models

Model	2-1(a)	2-1(b)	2-2(c)	3-0(d)	3-1(e)	3-2(f)	3-3(g)
Max noise (dB)	121	123	123	122	118	124	116

Comparing the sound pressure diagrams with the sound pressure level curves, we found that the selection of monitoring points was reasonable. The sound pressure value of the rear module was the largest, while the other parts were generally close to each other. The sound pressure values of 500 and 1000 Hz were big, but were small at 5000 Hz.

## Conclusions

2D and 3D asymmetric models were designed using engineering biomimetics, and optimised. We obtained optimised cross sections with good drag and noise reduction, and then added non-smooth features. The 2D model demonstrated great improvement, relative to an ordinary cylindrical bar, with a largest noise reduction of 60–80 dB. Then, by analysing the noise of the optimal asymmetric model at different speeds, we found that it had a very big effect on noise generation. For the asymmetric model at low speeds, the rear of the model produced a vortex street; however because of the low speed, the noise was not very loud. With increasing speed, the addition of semicircles to the model resulted in flow at the rear that did not produce a vortex street; again, the increase in noise levels was not significant. So, for high-speed conditions, an asymmetric non-smooth cross sectional shape is a better choice.

In the 3D model, we explored the influence on noise of increasing the V, and U-shaped slot and hemispherical non-smooth characteristics. It was found that the different non-smooth shapes of cross section had differing noise reduction effects. For the asymmetric cross sections of 3D model, the noise reduction effect of the V groove was not obvious. The U-shaped slot and hemisphere had a perfect noise reduction effect.

## Abbreviations

FW–H equation: Ffowcs-Williams–Hawkings equation
CFD: computational fluid dynamics
DES: detached eddy simulation
CAD: computer aided design
